# Chronic non bacterial osteitis- a multicentre study

**DOI:** 10.1186/s12969-018-0290-5

**Published:** 2018-11-22

**Authors:** Chandrika S. Bhat, Catriona Anderson, Aoibhinn Harbinson, Liza J. McCann, Marion Roderick, Adam Finn, Joyce E. Davidson, Athimalaipet V. Ramanan

**Affiliations:** 1 Departments of Paediatric Rheumatology and Immunology, Bristol Royal Hospital for Children, Bristol, BS2 8BJ UK; 2 Scottish Paediatric and Adolescent Rheumatology Network, NHS National Services Scotland, Meridian Court, 5 Cadogan Street, Glasgow, G2 6QE UK; 3 Alder Hey Children’s NHS Foundation Trust, East Prescott Street, Liverpool, L14 5AB UK; 4 Schools of Population Health Sciences and Cellular and Molecular Medicine, University of Bristol, Bristol, UK; 5 Bristol Medical School, University of Bristol, Bristol, UK; 6 Department of Paediatric Rheumatology, Level 6, Education Centre, Upper Maudlin Street, Bristol, BS2 8BJ UK

**Keywords:** Multifocal, Non-infectious osteitis, Auto inflammatory, Bristol diagnostic criteria, Whole body magnetic resonance imaging, Bisphosphonates

## Abstract

**Objective:**

To understand the demographics, clinical features and treatment outcomes of Chronic Non-bacterial Osteitis (CNO) from three tertiary paediatric rheumatology services in the United Kingdom.

**Methods:**

Children less than 18 years of age diagnosed with CNO between 2001 to 2016 from one tertiary service and between 2001 to 2017 from two tertiary services were included. Clinical notes were reviewed and all pertinent data were collected on a pre-defined proforma. One hundred and thirty one patients were included in the study. The Bristol diagnostic criteria were applied retrospectively.

**Results:**

Retrospective analysis of the data showed that the disease was more common in girls than boys (2.5:1), median age at onset of symptoms was 9.5 years (IQR 8 to 11 years). Bone pain was the predominant symptom in 118/129 (91.4%) followed by swelling in 50/102 (49.01%). Raised inflammatory markers were present in 39.68% of the patients. Whole body Magnetic Resonance Imaging (MRI) was a useful diagnostic tool. Metaphyses of long bones were most often involved and the distal tibial metaphyses 65/131 (49.6%) was the most common site. Non-steroidal anti-inflammatory drugs were used as first line (81.67%) followed by bisphosphonates (61.79%). Treatment was escalated to a TNF blocker when response to bisphosphonates was suboptimal. The disease was in remission in 82.4% of the patients during the last follow up.

**Conclusion:**

Our multicentre study describes features and outcomes of CNO in a large number of patients in the United Kingdom.

**Significance and innovation:**

- Raised inflammatory markers were present in 39.68% of our patients.
- Whole body MRI is useful for diagnosis and also determining response to treatment.
- A greater number of lesions were detected on radiological imaging compared to clinical assessment.
- Metaphyses of long bones were most often involved and the distal tibial metaphyses (49.6%) were the most common site.
- Non-steroidal anti-inflammatory drugs were used as first line (81.67%) followed by bisphosphonates (61.79%).
- There was no difference in number of medications used for management in unifocal versus multifocal disease.
- TNF blockers were used with good effect in our cohort.

## Introduction

Chronic nonbacterial osteitis (CNO) or chronic recurrent multifocal osteomyelitis (CRMO) is a rare auto inflammatory disorder characterised by the presence of sterile bone lesions [1]. The disease predominantly affects the metaphyses of long bones, pelvis, vertebrae and clavicles [2]. It is most commonly multifocal and recurrent. As unifocal and/or non-recurrent forms have also been described the term Chronic Nonbacterial Osteitis is considered to be more appropriate than CRMO [3]. A subset of CNO patients have inflammatory organ involvement and CNO is also associated with other auto inflammatory disorders like psoriasis, Crohn’s disease, ulcerative colitis, pustulosis and acne. CNO is considered to be the paediatric form of SAPHO syndrome that comprises Synovitis, Acne, Pustulosis, Hyperostosis and Osteitis [4]. A CNO susceptibility gene (FBLIM1) has recently been identified by whole genome sequencing in two unrelated patients from South Asia but the genetic susceptibility around CNO remains incompletely understood [5].

CNO is a rare disorder but advances have been made in understanding the clinical, histological and radiological features, in addition to long-term outcomes. Apart from the Eurofever registry that included 486 patients [6], most of the studies published so far have included relatively small numbers of children [3, 7–9]. We therefore conducted a retrospective study to improve our understanding of the clinical profile of the disease, optimal investigations, therapeutic options and the long-term outcome from three tertiary services in United Kingdom (UK).

## Methods

The medical records of patients with CNO from three tertiary services in United Kingdom were reviewed. All children < 18 years of age diagnosed as CNO by a paediatric rheumatologist after appropriate clinical, laboratory and radiologic investigations were included. Children diagnosed with CNO between 2001 to 2016 from one tertiary service and between 2001 to 2017 from two tertiary services were included. Data collected included demographic, clinical, laboratory, radiological and treatment characteristics.

Demographic details recorded were: gender, age of onset of symptoms, age and year of diagnosis, time taken to diagnosis and ethnicity. Clinical characteristics noted were: presenting symptoms (bone pain, swelling, fever, and other constitutional symptoms), initial diagnosis, preceding illness and symmetry of symptoms. The site of bone pain or bone swelling was also noted. The presence of other auto inflammatory conditions like psoriasis, inflammatory bowel disease (IBD), pustulosis and acne in the affected child and family were noted. On follow up, the number of flares and other complications experienced were recorded. Data collected for laboratory investigations included inflammatory markers - erythrocyte sedimentation rate (ESR) and C-reactive protein (CRP), and where available, anti nuclear antibody (ANA) and HLA-B27. Laboratory reference ranges were used for ESR and CRP. Histological parameters concentrated on the results of the bone biopsy for the presence of plasma cells, mononuclear cells, fibrosis and chronic inflammation. Microbiological cultures were also noted where available. Radiological evaluation included the imaging modality used i.e. plain radiograph, ultrasound scan, radionuclide bone scan, computed tomography scan (CT) or magnetic resonance imaging (MRI). The Bristol Diagnostic Criteria were applied retrospectively [10]. The criteria state that a diagnosis of CNO can be made in the presence of-

- Typical clinical and radiological findings in more than one bone (or clavicle alone) without significantly raised inflammatory markers OR
- Typical clinical and radiological findings in one bone plus inflammatory changes (plasma cells, osteoclasts, fibrosis or sclerosis) on bone biopsy with no bacterial growth.

Typical clinical findings include bony pain with or without localised swelling and absence of significant local or systemic features of inflammation or infection. Typical radiological findings constitute plain x-rays showing a combination of lytic areas, sclerosis and new bone formation and MRI, preferably Short T1 Inversion Recovery sequences (STIR), showing bone marrow oedema, bone expansion, lytic areas and/or periosteal reaction.

Features of treatment recorded were drugs used, where documented whether their use was followed by any apparent clinical or radiological improvement and the number of drugs needed to induce remission. Remission was described as clinical or radiological. Clinical remission meant subsidence of pain, swelling or constitutional symptoms. Radiological remission was defined as reduction of activity or reduction in number of radiological lesions. Post treatment imaging was usually performed in those who received pamidronate or a TNF blocker.

We stratified our cohort based on median age of onset of symptoms, sex, number of sites of involvement, year of diagnosis and studied disease phenotype in each subgroup. Statistical analysis was performed using Microsoft Excel version 12.0 and t-Test was used to evaluate significance of differences. Results were expressed as median and interquartile range (IQR) for continuous variables and as number (%) for categorical variables.

## Results

Data were collected on 131 children from 8 paediatric centres. This included 4 tertiary centres (Bristol, Liverpool, Edinburgh, and Glasgow) and also District General Hospitals within the Scottish SPARN network.

### Demographics

94/131 (71.8%) were female. The median age at onset of symptoms was 9.5 years (IQR 8 to 11 years) median age at diagnosis was 10.7 years (IQR 8.9 to 12.7 years). The median time to diagnosis was 12 months (IQR 5 to 24 months). Other baseline characteristics are summarised in Table 1. The youngest patient was 23 months old.

**Table 1:** Baseline characteristics of patients with CNO

CHARACTERISTICS	FEATURES	NUMBER (%)
Age at diagnosis (median;IQR years)		10.7 (8.9 to 12.7)
Gender F/M;ratio		94/37;2.54
Clinical Characteristics	Bone pain (*n* = 129)	118 (91.4%)
	Swelling (*n* = 102)	50 (49.01%)
	Fever (*n* = 79)	6 (7.59%)
	Symmetrical symptoms (*n* = 125)	24 (19.2%)
	Clinical unifocal involvement	92 (70.22%)
	Synovitis	9 (6.87%)
	Arthritis	9 (6.87%)
	Hyperostosis	43 (32.82%)
Number of sites	Clinical	1.29 (range 0–3)
	Radiological	3.27 (range 1–13)
Extraosseous involvement	Psoriasis	5 (3.87%)
	Ulcerative colitis	1 (0.76%)
	Pustulosis	10 (7.63%)
Investigations	Raised inflammatory markers (*n* = 126)	50 (39.68%)
	ANA positive (*n* = 26)	3 (11.53%)
	HLA B27 positive (*n* = 15)	1 (6.66%)
	Bone biopsy performed	73 (55.72%)
	Positive culture (*n* = 3) from bone biopsy	Coagulase negative staphylococcus: 2 (66.66%)
		*Staphylococcus aureus*: 1 (33.33%)
Family history of-	Psoriasis (*n* = 130)	20 (15.38%)
	Inflammatory bowel disease (*n* = 130)	4 (3.07%)
	Autoimmune disorders (*n* = 130)	6 (4.61%)

### Investigations

Laboratory investigations revealed a raised ESR (range < 1 to 148 mm/hr) or CRP (range < 1 to 400 mg/L) in 50/126 (39.68%) of the patients. ESR was mildly elevated (< 50 mm/hr) in 32/42(76.1%), moderately elevated (50 to 100 mm/hr) in 8/42(19.04%) and highly elevated (> 100 mm/hr) in 2/42(4.75%). CRP was mildly elevated (< 50 mg/L) in 16/28(57.14%), moderately elevated (50 to 100 mg/L) in 8/28(28.6%) and highly elevated (> 100 mg/L) in 4/28(14.28%). Both ESR and CRP were raised in 20 patients and 11 had proportionate elevation of both. One patient had unusually high inflammatory markers (CRP = 400, ESR = 100) at time of presentation associated with fevers and widespread joint pain. The diagnosis of CNO was made after negative tests for infection, typical features of CNO on extensive imaging (X rays, bone scan, CT and MRI), inadequate response to antibiotics and, in contrast, an immediate and sustained response to bisphosphonate treatment.

Bone biopsy was performed when diagnosis was uncertain and to exclude malignancy. 73/131(55.72%) patients underwent a bone biopsy.19/73 (26.02%) had a ‘chronic inflammatory infiltrate’, 15/73 (20.54%) had evidence of fibrosis, 13/73 (17.80%) had plasma cell infiltrate and 11/73 (15.06%) had a mononuclear cell infiltrate. A negative culture result was obtained in 70/73 (95.89%) patients. Of the three positive culture results, coagulase negative staphylococcus (CONS) was isolated in two patients and *Staphylococcus aureus* from one patient on enrichment culture. All three failed to respond to antibiotic treatment and further imaging with a WB-MRI scan revealed multifocal signal changes thereby confirming a diagnosis of CNO. Bone biopsy was repeated in the child with *Staphylococcus aureus* and was negative. Organisms isolated initially were most likely contaminants.

**Fig. 1:**
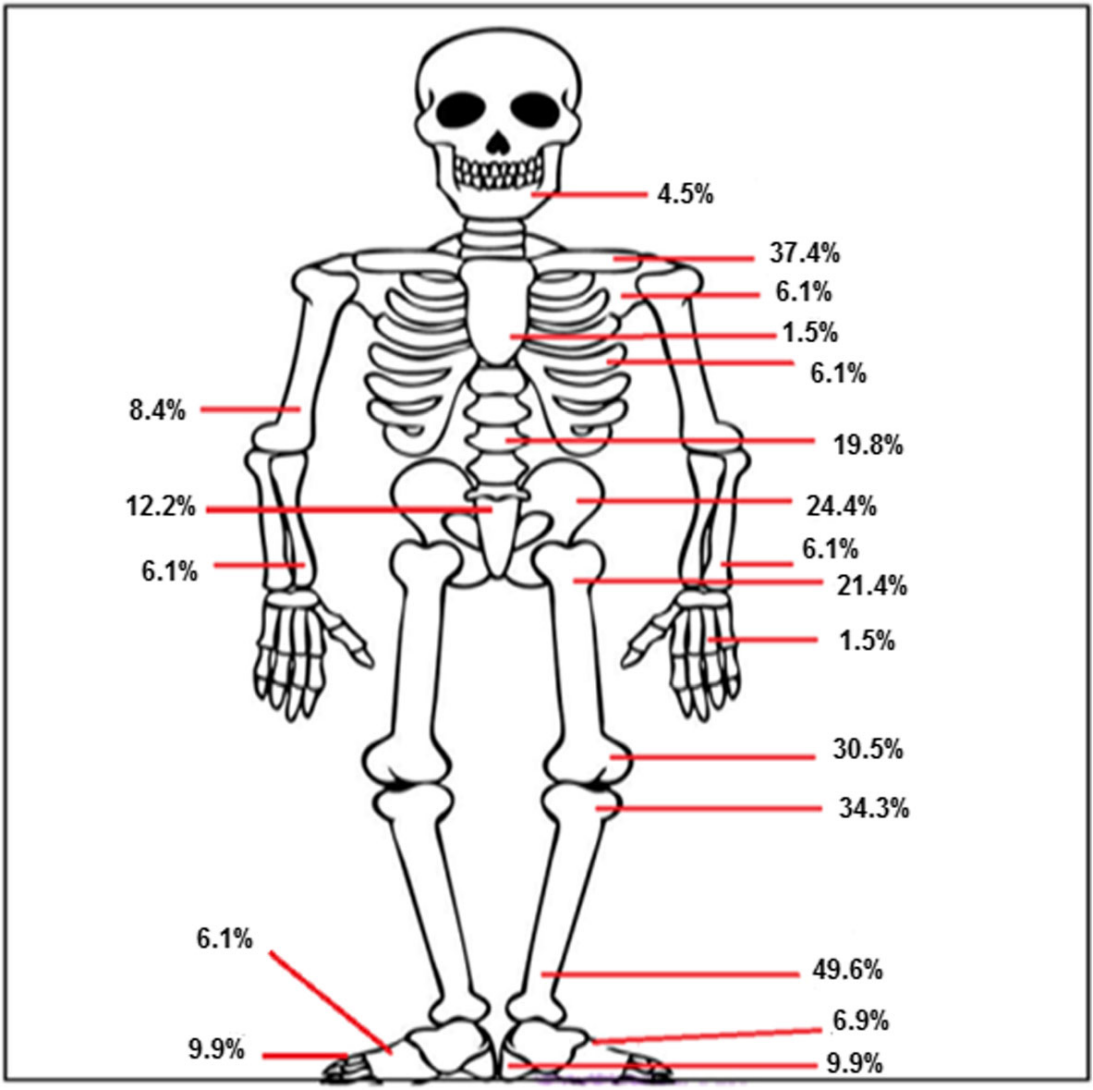
Sites of involvement in CNO

Plain radiographs were performed in 104/131 (79.4%) patients. Where available images were reviewed. Sclerosis was reported in 44/85 (51.76%) patients, periosteal reaction in 33/85 (38.82%) and lytic lesions in 24/85 (28.23%).Ultrasound scans were used for evaluation of joint swelling, superficial bone swelling or screening of the abdomen in 29/131(22.13%). 34/131 (26%) of the patients had a radionuclide bone scan. An increase in uptake was demonstrated in areas of active disease. A CT scan was done in 24/131 patients (18.32%). Expansion of bone was reported in 11/24(45.83%), sclerosis in 7/24 (29.16%), lytic lesions 6/24 (25%) and periosteal reaction in 6/24 (25%) patients. A whole body MRI was performed in 122/131(93.12%) patients. 429 lesions were detected with the help of all imaging done and 405 lesions with the use of a whole body MRI. The most common site of involvement was the distal tibial metaphyses 65/131(49.61%). Other sites of involvement on radiological imaging have been illustrated in Fig. 1. Unifocal disease at presentation was seen in 22/131(16.79%) patients. The clavicle was the most common site for unifocal disease in 12/22 (54.54%). The sacroiliac joint was involved in 9/131(6.87%) patients.

### Treatment

NSAIDs were used as first line in treatment of 107/131(81.67%) patients followed by bisphosphonates in 89/131(67.93%). Pamidronate was the preferred bisphosphonate in all three services. TNF blockers were used when patients failed other agents but not as first line in any of the patients, including those with systemic symptoms at onset of disease. Adalimumab was the preferred TNF blocker and was used in 11/131(8.39%) patients. Infliximab was commenced in 8/131(6.10%) patients. Psoriasis was reported in 2/8(25%) patients treated with infliximab. Both these cases had a family history of psoriasis but did not have any signs until they were treated with infliximab. Etanercept was used in one patient with good effect. Drugs and their observed response rates based on the judgement of the treating clinician have been summarised in Table 2. 21/26 (80.7%) patients with vertebral disease and 4/6 (66.7%) with mandibular involvement received bisphosphonate as first line therapy. Osteonecrosis of the jaw was not reported in any case.

**Table 2:** Observed response rates to treatment and outcome of disease

Drug	Number of patients used (%) (*n* = 131)	Number of patients who responded	Observed response rate (%)
NSAIDs	107 (81.6)	53/92	57.6
Bisphosphonates	89 (67.93)	61/89^a^	68.53
		55/89^b^	61.79
Methotrexate	18 (13.74)	7/16	43.75
Corticosteroids	13 (9.92)	8/10	80
Adalimumab	11 (8.39)	10/11	90.9
Infliximab	8 (6.10)	7/8	87.5
Sulfasalazine	3 (2.29)	2/3	66.66
Etanercept	1 (0.76)	1/1	100
Mesalazine	1 (0.76)	1/1	100

^a^clinical remission

^b^clinical and radiological remission

We segregated our cohort based on sex, age of onset of symptoms (< 10 years vs. > 10 years), unifocal vs. multifocal disease and year of diagnosis (before or after 2009) and studied disease characteristics in each group.

#### Sex

There was little observed difference in the number of lesions *(3.3* versus *2.9) (p = 0.13).* However the number of drugs required to induce clinical or radiological remission in our cohort was higher in girls than boys *(2.1* versus *1.8)* (*p = 0.03*). Episodes of flares were commoner in females (*64.1%)* than males (*43.8%*) (*p = 0.02*).

#### Age of onset

The percentage of girls was greater in the group that developed symptoms after 10 years of age *(76.1%* versus *72.1%)*. There was no evidence of a difference in the number of lesions between the two age groups *(3.13* versus *3.18; p = 0.44)* nor the number of drugs required to induce remission *(1.93* versus *2.08; p = 0.18)* nor the number of painful flares experienced *(0.24* versus *0.25; p = 0.31).*

#### Unifocal versus multifocal disease

The number of drugs required to induce remission was 2.2 with unifocal disease and 2.0 with multifocal disease *(p = 0.16)*. Inflammatory markers were raised in 38.1% of the patients with unifocal disease and 40.0% with multifocal disease *(p = 0.56)*.

#### Year of diagnosis (before or after 2009)

The median time to diagnosis before 2009 was 16 months (IQR 5 to 27 months) and 11 months (IQR 5.25 to 24 months) after 2009. There was no difference in the number of patients who had a bone scan *(37.1%* vs *21.1%; p = 0.06),* WB MRI *(91.4%* vs *96.7%;p = 0.10)* or treatment with pamidronate *(57.1%* vs *74.4%;p = 0.06)*. However, there was a significant difference in the number of patients who received antibiotic therapy *(42.8%* vs *21.2%; p = 0.01)* or methotrexate *(22.8%* vs *8.9%; p = 0.03)* before and after 2009.

### Follow up

On follow up, 75/131 (57.3%) patients reported painful flares whilst on or following completion of treatment. 6/131(4.6%) patients were managed for chronic pain. One patient developed a leg length discrepancy thought to be due to CNO. Other complications included vertebral compression fractures in two patients and a metatarsal fracture in one patient. Outcomes are summarised in Table 3. 30/131 (22.9%) were transitioned to adult services.

**Table 3:** Outcome of disease at last follow up

Outcome	Number	Percentage
Disease remission	108	82.4
Painful flares	75	57.25
Unifocal recurrent disease (*n* = 16)	2	12.5
Unifocal non recurrent disease(*n* = 16)	14	87.5
Multifocal recurrent disease(*n* = 109)	21	19.26
Multifocal non recurrent disease(*n* = 109)	88	80.73

## Discussion

In our study from three UK tertiary services, the demographic characteristics of our patients are similar to those reported in previously published studies [7, 9]. The initial symptom of bone pain was reported mainly in the lower limbs (54.3%) similar to previous studies [8, 9]. Associated auto inflammatory conditions included IBD (*n* = 1) and psoriasis (*n* = 5) and were less common than previously reported [8]. In relation to this, inflammatory markers were raised in only 39.6% of our patients in contrast to previous studies where CRP and/or ESR levels were increased in 50 to 90% [4, 7]. One patient tested positive for HLA B27 but did not have features of ERA. However the majority of patients in this cohort were not tested for HLA B27 so this observation must be interpreted with caution. Seven patients progressed to develop ERA of whom only one was tested for HLA B27, which was negative. In a previously published study none of the children who evolved to a spondyloarthropathy were HLA B27 positive [11].

The mean number of painful sites reported clinically was less than the mean number of radiological sites detected on imaging (1.3 vs. 3.3) with 64.3% lesions being asymptomatic. This highlights the importance of imaging in detecting asymptomatic lesions. A whole body MRI is potentially more sensitive than other imaging modalities in identifying lesions at diagnosis and also in assessing response to treatment. It is also preferable to a radionuclide bone scan or CT scan since it avoids exposure to radiation. The distal tibial metaphysis (49.6%) was the most commonly involved site in our group. Our results are concordant with other studies where the tibia was the most commonly affected bone [3, 9].

This study used the Bristol Diagnostic Criteria, which were applied retrospectively. With their use 23/73 (31.5%) of biopsies could have been avoided. Previously Jansson et al. also published diagnostic criteria and a clinical score to aid diagnosis of CNO [12]. Neither tool has been evaluated in unrelated cohorts or is internationally accepted, but the routine use of diagnostic criteria may aid an early diagnosis and avoid unnecessary investigations.

In our cohort NSAIDs were the preferred first line agent except patients with mandibular or vertebral involvement in whom bisphosphonates were usually used as first line. Treatment was escalated to a TNF blocker when the response to bisphosphonates was suboptimal. TNF blockers were used with good effect but the observed response rates documented in Table 3 need to be interpreted with caution due to low patient numbers. Tendency to use antibiotics or methotrexate was less common after the year 2009. This could be attributed to increased disease awareness and evolution of treatment options over the past ten years.

Treatment practices for CNO are variable worldwide with a tendency to use TNF blockers more commonly than bisphosphonates in North America [13]. Access to TNF blockers has been variable in UK for this indication. The Childhood Arthritis and Rheumatology Research Alliance (CARRA) have developed three Consensus Treatment Plans for the treatment of CNO in patients refractory to NSAIDs and/or with active spinal lesions with either methotrexate or sulfasalazine, TNF blockers (with or without methotrexate) or bisphosphonates. Use of these Consensus Treatment Plans will provide more information on efficacy in the absence of randomised control trials [14].

On follow up, the disease was in remission in 82.4% of patients while 17.6% had polycyclic disease. Traditionally, the long term clinical outcome for children with CNO was thought to be good but recent studies have demonstrated significant long term morbidity [15].

A limitation of our study is that it is a retrospective analysis and data collection was not entirely homogeneous across the three tertiary services. The preferred diagnostic modalities and therapeutic options varied between the three centres and response to treatment was based on the interpretation of the treating clinician. Due to the retrospective design, missing data were inevitable, particularly for ANA and HLA B27 results.

## Conclusion

From our study we conclude that CNO is a chronic disease with significant disease-related sequelae in a subset of patients. The age of disease onset did not have a major impact on the severity of disease. Whole body MRI is a useful tool in detecting asymptomatic lesions. Vertebral and mandibular involvement warrants aggressive treatment. The outcome of the disease with the use of appropriate treatment is fairly good. Increased awareness of this disease amongst clinicians might hasten diagnosis and improve treatment outcomes. Future studies including more patients from additional tertiary centres are required to formulate standard definitions, consolidated investigation pathways and treatment strategies for patients with CNO. One of the strengths of our series is that this is a cohort of patients from three large services and therefore is likely to be representative of the true spectrum of disease in United Kingdom but this needs to be evaluated in larger patient numbers.

## Abbreviations

CNO: Chronic non-infectious osteomyelitis
CRMO: Chronic recurrent multifocal osteomyelitis
CRP: C reactive protein
CT: Computed Tomography Scans
ERA: Enthesitis Related Arthritis
ESR: Erythrocyte Sedimentation Rate
STIR: Short T1 Inversion Recovery sequences
WB MRI: Whole body MRI
